# Editorial: Methods in skin cancer

**DOI:** 10.3389/fonc.2023.1150450

**Published:** 2023-03-03

**Authors:** Rosario Caltabiano

**Affiliations:** Department of Medical and Surgical Sciences and Advanced Technologies “G.F. Ingrassia” Anatomic Pathology, University of Catania, Catania, Italy

**Keywords:** skin, cancer, histopathology, methods, review

## Introduction

One of the crucial topics of the research in the field of dermatology has always been to make the diagnosis of both inflammatory and neoplastic skin diseases as non-invasive as possible (1–3). To this purpose, several techniques such as dermoscopy, reflectance confocal microscopy (RCM) and line-field confocal optical coherence tomography (LC-OCT) have been introduced in clinical practice. These tools allow to obtain a better visualization of cutaneous lesions both horizontally and vertically, guaranteeing a more sensitive and specific clinical diagnosis than clinical examination alone (4–7). The diagnostic reliability of these methods has been proved by several studies in which their findings have been compared with those obtained by confirmatory biopsies followed by histological examination (8–15); in this regard, a strong correlation with the histopathologic features of several cutaneous disorders has been reported in literature, emphasizing that these non-invasive methods could serve in the future as potential histological surrogates, avoiding the patient sometimes unnecessary biopsies (8–15).

Both vertical and horizontal high-quality images of a skin lesion from the stratum corneum to the papillary dermis are provided by RCM and LC-OCT but, while the former only allows a two-dimensional visualization of the skin, the latter can emit a three-dimensional reconstruction of the tissue (4–7). RCM and LC-OCT have been validated as reliable diagnostic tools in several cutaneous diseases and are currently used by many dermatologists in their clinical practice (4–7).

In addition to the above-mentioned techniques, novel experimental methods are progressively being introduced and the aim of the present Research Topic collection was to highlight the latest experimental techniques and methods used to investigate fundamental questions in Skin Cancer and their clinical application. Four studies (three “original research” articles and one “case report”) are included (Song et al., Han et al., Ross et al., Lv et al.). In the first article, Song et al. studied the relationship between hypercoagulable state and prognosis in cutaneous melanoma. They developed a prognostic model based on seven coagulation-related genes (CRGs) (*ANG, C1QA, CFB, DUSP6, KLKB1, MMP7* and *RABIF*) and correlated it with clinico-pathologic features, tumor microenvironment and response to immunotherapy. The authors found that cutaneous melanoma prognosis could be predicted by this model, as longer overall survival times were seen in the low-risk group than in the high-risk group, and better response rates to the immunotherapy was found in the low-risk group. A similar topic is covered in the second Original Research article by Han et al. These authors developed a prognostic model of cutaneous melanoma, based on the expression of some immunogenic cell death (ICD)-related genes, to predict cutaneous melanoma outcome and potential response to immunotherapy. Two risk groups (high-risk and low-risk) were identified and ICD-related gene expression was found to be lower in the high-risk group than in the low-risk group, while the survival times were longer in the low-risk one.

The relevance of these studies lies in the fact that the authors were able to find factors potentially capable of predicting the prognosis of a frequently metastasizing malignancy, for whose diagnosis and prognosis few biomarkers are currently available.

The last Original Research article focuses on the treatment of cutaneous basal cell carcinoma (BCC) (Ross et al.). BCC is the most frequent form of skin cancer and is traditionally considered an almost indolent neoplasm, being its biological behavior characterized by low rates of local recurrence and sporadic cases of distant metastases (Ross et al.). Surgical excision is the most frequent therapeutic approach, although Mohs surgery or curettage and electrodessication are also used. Ross et al. proposed the application of ultrashort electric pulses during Nano-Pulse Stimulation™ (NPS™) therapy as a novel treatment option for BCCs, especially for lesions located at the face or at non-surgically attackable sites. It has been demonstrated that NPS™ therapy stimulates programmed cell death of tumor cells, reducing the risk of scarring. The authors performed this therapy on a prospective series of 37 BCCs which underwent surgical excision followed by histological examination after 60 days from the treatment. They found that NPS™ therapy was extremely effective in eradicating BCC lesions, leading to a complete histologic clearance of the tumor in about 90% of the patients. In addition, minimal scarring was found in these histologic samples, emphasizing that NPS™ therapy could be an emerging therapeutic option especially for those BCC lesions whose complete excision is not easily achievable.

Finally, Lv et al. reported an unusual case of trichilemmal carcinoma (TC) of the abdomen, associated with axillary and inguinal lymph node metastases and treated by surgery and local radiotherapy. Although TC is considered a biologically slow-growing tumor, its clinical course may be rarely characterized by local recurrences and distant metastases; due to the rarity of these occurrences, there are still no standardized treatments. The case presented by the authors underwent surgical excision and adjuvant radiotherapy and the patient did not exhibit local recurrence or metastasis at one year of follow-up. This study is relevant as it suggests regional lymph node dissection followed by local radiotherapy as a potential therapeutic option for patients affected by TC metastatic to lymph nodes.

We thank all the authors and the reviewers who contributed to this Research Topic. We also wish that all the readers of this Research Topic will enjoy the published manuscripts.

## Author contributions

The author confirms being the sole contributor of this work and has approved it for publication.
